# Aspirin Inhibition of Group VI Phospholipase A2 Induces Synthetic Lethality in AAM Pathway Down-Regulated Gingivobuccal Squamous Carcinoma

**DOI:** 10.3390/cells11010123

**Published:** 2021-12-30

**Authors:** Kshama Pansare, Bhabani Mohanty, Ranjeeta Dhotre, Aafrin M. Pettiwala, Saili Parab, Neha Gupta, Poonam Gera, Nilesh Gardi, Rucha Dugge, Priyanka Sahu, Ruby Alhans, Pradnya Kowtal, Pradip Chaudhari, Rajiv Sarin

**Affiliations:** 1 ICGC Lab, Advanced Centre for Treatment, Research and Education in Cancer, Tata Memorial Centre, Kharghar, Navi Mumbai 410210, India; kshamaj99@gmail.com (K.P.); ranjeeta.sd79@gmail.com (R.D.); aafrinmpettiwala9@gmail.com (A.M.P.); nehagupta.01592@gmail.com (N.G.); pgera@actrec.gov.in (P.G.); duggerucha@gmail.com (R.D.); priyanka.sahu0123@gmail.com (P.S.); rubyalhans@gmail.com (R.A.); pkowtal@actrec.gov.in (P.K.); 2 Small Animal Imaging Facility, Advanced Centre for Treatment, Research and Education in Cancer, Tata Memorial Centre, Kharghar, Navi Mumbai 410210, India; bhabani.bmohanty@gmail.com (B.M.); pchaudhari@actrec.gov.in (P.C.); 3 Biorepository, Advanced Centre for Treatment, Research and Education in Cancer, Tata Memorial Centre, Kharghar, Navi Mumbai 410210, India; sailisparab.30@gmail.com; 4 Department of Medical Oncology, Tata Memorial Hospital, Tata Memorial Centre, Mumbai 400012, India; nilesh.gardi2688@gmail.com; 5 Homi Bhabha National Institute, Training School Complex, Anushakti Nagar, Mumbai 400085, India; 6 Sarin Lab, Advanced Centre for Treatment, Research and Education in Cancer, Tata Memorial Centre, Kharghar, Navi Mumbai 410210, India

**Keywords:** phospholipase, PLA2G6, aspirin, arachidonic acid metabolism pathway, gingivobuccal squamous cell carcinoma

## Abstract

Background: To elucidate the role of iPLA2/PLA2G6 in gingivobuccal squamous cell carcinoma (GB-SCC) and to ascertain the synthetic lethality-based chemoprevention role of aspirin in arachidonic acid metabolism (AAM) pathway down-regulated GB-SCC. Methods: The in vitro efficacy of aspirin on GB-SCC cells (ITOC-03 and ITOC-04) was assessed by cell proliferation, colony formation, apoptosis, cell migration, cell cycle assay and RNA-seq, while inhibition of PLA2G6 and AAM pathway components was affirmed by qPCR, Western blot and immunofluorescence staining. The in vivo effect of aspirin was evaluated using NOD-SCID mice xenografts and immunohistochemical analysis. Results: We found that aspirin, which has been reported to act through the COX pathway, is inhibiting PLA2G6, and thereby the COX and LOX components of the AAM pathway. The findings were validated using PLA2G6 siRNA and immunohistochemical marker panel. Moreover, a pronounced effect in ITOC-04 cells and xenografts implied aspirin-induced synthetic lethality in the AAM pathway down-regulated GB-SCC. Conclusions: This study reveals that aspirin induces the anti-tumor effect by a previously unrecognized mechanism of PLA2G6 inhibition. In addition, the effect of aspirin is influenced by the baseline AAM pathway status and could guide precision prevention clinical trials of AAM pathway inhibitors.

## 1. Introduction

Gingivobuccal squamous cell carcinoma (GB-SCC) is the most common cancer in Southeast Asia where smokeless tobacco use is highly prevalent. GB-SCC is often diagnosed in late stages and has a poor prognosis despite substantial progress in treatment [1,2,3]. Therefore, it is important to discover novel approaches for early detection, prevention and management of GB-SCC [4]. 

Phospholipase A2 (PLA2) enzymes exert their action by hydrolyzing the sn-2 acyl bond of phospholipids and thereby releasing free fatty acids and lysophospholipids. Different PLA2 enzymes are involved in lipid metabolism and play a critical role in tumor progression [5]. In the arachidonic acid metabolism (AAM) pathway, phospholipids are hydrolyzed by cytosolic phospholipase (cPLA2) and intracellular calcium independent phospholipase (iPLA2) into arachidonic acid (AA), a 20-carbon unsaturated fatty acid. AA is further metabolized via cyclooxygenase (COX), lipoxygenase (LOX) and cytochrome P450 (CYP450) pathway to prostaglandins (PG) and thromboxanes A2 (TXA_2_); leukotrienes, lipoxins and hepoxillins; and hydroxyeicosatetraeonic acids (HETEs), epoxyeicosatrieonic acids, and ω-HETEs, respectively [5,6]. Eicosanoids such as PGE_2_ and TXA_2_ are inflammatory mediators known to be involved in cancer progression and angiogenesis, respectively [7]. Inflammation, one of the hallmarks of cancer promotes tumorigenesis through various mechanisms which manifest as infiltration of immune cells, excessive production of eicosanoids, cytokines, oxidants, matrix metalloproteinase, fibrosis, tissue damage and increased angiogenesis [8,9,10,11]. Furthermore, amongst the class of PLA2 enzymes, iPLA2 are the least studied and understood in the context of cancer, especially in GB-SCC [12]. Considering the pivotal role of inflammation in cancer, understanding the underlying molecular mechanisms of iPLA2, phospholipase A2 group VI (PLA2G6) in AAM pathway-mediated tumorigenesis is imperative.

Aspirin, a non-steroidal anti-inflammatory drug (NSAID) exerts its antineoplastic effect by blocking prostaglandin synthesis via inhibition of cyclooxygenase (COX-1, COX-2), a component of the AAM pathway that mediates or modulates inflammatory response [13]. The chemopreventive effect of aspirin has been reported in several cancers, especially colorectal cancer [13,14,15,16,17,18,19]. However, the chemopreventive or chemotherapeutic effects of aspirin have not been studied in GB-SCC, which is the most common smokeless tobacco-associated cancer. The anti-neoplastic effect of aspirin has shown to be via COX-dependent or independent mechanisms [20,21,22,23,24,25,26]. Earlier study has shown that aspirin can prevent HCC by targeting P4HA2 through NF-ĸB and suppressing collagen deposition [27]. It is important to study the chemopreventive mechanism of aspirin in different cancers which employ different pathways for cancer development and progression. Moreover, it is also plausible that in the presence of certain pre-existing deregulation of specific pathways, aspirin could induce synthetic lethality. 

In our International Cancer Genome Consortium (ICGC) Indian cohort, we have earlier reported a correlation between somatic loss-of-function mutations in the AAM pathway genes with improved survival in GB-SCC patients [28]. To understand the role of iPLA2/PLA2G6 in GB-SCC and to ascertain the chemoprevention and antitumor effect of aspirin, we performed a comprehensive analysis on two GB-SCC cell lines which we had earlier developed and characterized with exome and transcriptome analysis and identified a significantly down-regulated AAM pathway in one of them (ITOC-04) [29]. On the basis of our preliminary findings, we postulated that aspirin may exert its action via iPLA2 and have greater antitumor effect or require a lower dose for similar effect due to synthetic lethality in cancer cells with a baseline down-regulated AAM pathway.

## 2. Materials and Methods

### 2.1. Cell Lines and Reagents

ITOC-03 (RRID:CVCL_WS68) and ITOC-04 (RRID:CVCL_WS69) GB-SCC cell lines were maintained in Iscove’s Modified Dulbecco’s Medium (Gibco, Waltham, MA, USA) supplemented with 10% fetal bovine serum (Gibco, NY, USA) and antibiotic (InvivoGen, San Diego, CA, USA) at 37 °C in a humidified atmosphere containing 5% CO_2_. Aspirin (Sigma-Aldrich, Darmstadt, Germany) was dissolved in ethanol to a stock concentration of 10 mg/mL. 

### 2.2. Cell Viability Assay

ITOC-03 and ITOC-04 cells were seeded in 96-well plates at a density of 1 x 10^4^ cells/well. Cells were treated with aspirin at concentrations of 0, 2.5, 5 and 10 mmol/L and incubated for different time intervals at 37 °C, 5% CO_2_. MTT assay was performed as described earlier [29]. 

### 2.3. Colony Formation Assay 

A total of 1 × 10^3^ cells/well were seeded in a 6-well plate and treated with 0, 2.5 and 5 mmol/L aspirin for 48 h, and then cultured for 10 days. Colonies were washed with 1X phosphate buffered saline, fixed in methanol and stained with 0.1% crystal violet solution. Colonies comprising more than 50 cells were counted. 

### 2.4. Immunofluorescence Staining

Immunofluorescence staining was performed as described earlier [29]. Cells were incubated with specific primary antibody—Ki-67 (Mouse monoclonal, Cell Signaling, Beverly, MA, USA, 9449, 1:200), TBXAS1 (Mouse monoclonal, Abcam, MA, USA, ab119057, 1:100), followed by incubation with secondary antibody (Alexa Fluor 568 anti-mouse IgG (H + L), Molecular Probes, Waltham, MA, USA, A11004). Quantification of signal intensity was performed with the LSM Image browser (Carl Zeiss). Briefly, ROI were defined around nuclear or cell boundaries of each cell followed by extraction of signal intensities of DAPI and respective fluorescence channels. Relative mean intensity was calculated as a ratio of marker/DAPI-associated fluorescence. Quantification was performed on 50 nuclei for each sample. 

### 2.5. Cell Cycle Analysis

A total of 1 × 10^6^ cells/well were seeded in a 6-well plate and treated with 0, 2.5 and 5 mmol/L aspirin. After 48 h, cells were harvested, rinsed with 1X PBS, fixed in 70% ethanol for 60 min, incubated with 100 µg/mL RNase A (Sigma-Aldrich, Darmstadt, Germany) and stained with 50 µL propidium iodide (Sigma-Aldrich, Darmstadt, Germany) for 30 min at 37 °C. Acquisitions were performed on BD FACSCalibur (Becton Dickinson, San Jose, CA, USA) and data analyzed using the Modfit software (version 2.0). 

### 2.6. Annexin V and Propidium Iodide Staining

Apoptotic cells were detected using FITC Annexin V apoptosis detection kit I (BD Biosciences, NJ, USA) according to the manufacturer’s instructions. Briefly, 1 × 10^6^ cells/well were seeded in a 6-well plate and treated with 0, 2.5 and 5 mmol/L aspirin. After 48 h, cells were harvested, rinsed with PBS and stained with Annexin V-FITC and propidium iodide. The stained cells were detected using BD FACSCalibur (Becton Dickinson, San Jose, CA, USA) and data analyzed using the FlowJo software (version 10.0). 

### 2.7. Transmission Electron Microscopy (TEM)

Ultra-structure analysis was carried out on ITOC-03 and ITOC-04 cell lines treated with 0, 2.5 and 5 mmol/L of aspirin. TEM was performed as described earlier [29].

### 2.8. Scratch Assay

A total of 2 x 10^6^ cells/well were seeded in a 6-well plate and treated with 0, 2.5 and 5 mmol/L aspirin. The wound was inflicted with a pipette tip and images were captured every 8 h on an Olympus IX51 microscope (Olympus, Hamburg, Germany) and analyzed using the ImageJ software. The relative cell free area at respective time points was calculated by considering the inflicted wound at 0 h as 100%.

### 2.9. siRNA Transfection 

A total of 1 x 10^6^ cells/well were transfected with 10 nM PLA2G6 siRNAs and scrambled siRNA using DharmaFECT transfection reagent (Dharmacon, Laffayette, LA, USA) as per the manufacturer’s instructions. After 48 h, cells were harvested, rinsed with PBS and subjected to RNA extraction. siRNA sequences are specified in Table S1.

### 2.10. RT-PCR and qRT-PCR 

Total RNA was isolated from untreated and aspirin-treated cells using the miRNeasy mini kit (Qiagen, Hilden, Germany). cDNA synthesis was performed using the RevertAid RT kit (Thermo Scientific, Waltham, MA, USA), followed by RT-PCR. qRT-PCR was performed using the Quantstudio 12K flex (ABI, Carlsbad, CA, USA) and Power SYBR Green PCR Master Mix (ABI, Warrington, UK). A total of 10 pM of each primer and 10 ng of cDNA was used in qPCR reaction. Primer sequences are specified in Table S2.

### 2.11. Immunoblotting

Total protein extracts of 50 μg were resolved on precast 4–12% SDS-PAGE (Invitrogen, Waltham, MA, USA) gels and transferred onto PVDF membrane (GE Healthcare, Darmstadt, Germany). Ponceau S staining was performed to confirm transfer of proteins. The detailed protocol with antibody details is provided in Supplementary Materials & Methods. 

### 2.12. RNA Sequencing

Total RNA was isolated from untreated and 5 mmol/L aspirin-treated ITOC-03 and ITOC-04 cells using miRNeasy mini kit (Qiagen, Hilden, Germany). RNA libraries were constructed using the TruSeq RNA sample preparation kit (Illumina, San Diego, CA, USA) according to the manufacturer’s instructions. The untreated and aspirin-treated samples were sequenced on the HiSeq 2500 platform (Illumina, San Diego, CA, USA) with paired-end reads. Detailed RNA-seq analysis is provided in Supplementary Materials & Methods.

### 2.13. Mouse Xenograft and Positron Emission Tomography Imaging

The animal study was approved by the Institutional Animal Ethics Committee (Project no. 10/2016). All animal experiments were performed in accordance with relevant guidelines and regulations of the Institutional Animal Ethics Committee. The detailed protocol is provided in Supplementary Materials & Methods.

### 2.14. Immunohistochemical Analysis

Protein expression of Ki-67, NF-ĸB, GGT7, PLA2G6, COX-2, TBXAS1 was analyzed by IHC using horseradish peroxidase polymer-based detection kit (Envision Plus, Dako, Glostrup, Denmark). The detailed protocol with antibody details is provided in Supplementary Materials & Methods.

### 2.15. Statistical Analysis

All experiments were performed in triplicates. The data are presented as mean ± SEM. The differences between different treatment groups were analyzed using One-way analysis of variance (ANOVA) with the Bonferroni post-test and Student’s t test. The statistically significant differences were considered as per the following criteria: * *p* < 0.05, ** *p* < 0.01, *** *p* < 0.001.

## 3. Results

We had previously reported comprehensive genomic characterization [29] and key differences in the genetic alterations and expression changes in different pathways in these two cell lines (Table S3). In vitro and in vivo studies were carried out on these two GB-SCC cell lines with (ITOC-04) or without (ITOC-03) down-regulated AAM pathway. 

### 3.1. Higher Inhibitory Effect of Aspirin on Cell Viability and Colony Formation in AAM Pathway Down-Regulated Cell Line

**Figure 1:**
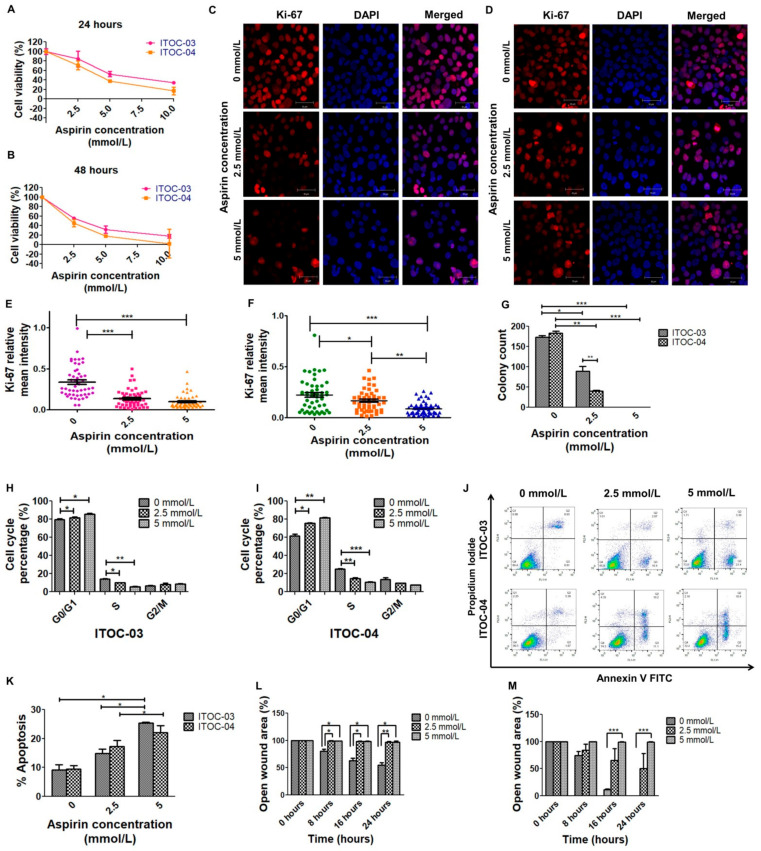
Aspirin inhibits cell viability, cell proliferation rate, colony formation, cell migration and induces cell cycle arrest and apoptosis in GB-SCC cells. (**A**,**B**) Cell viability assay showing dose- (0 mmol/L, 2.5 mmol/L, 5 mmol/L and 10 mmol/L) and time-dependent (24 h, 48 h) inhibition of ITOC-03 and ITOC-04 cells on treatment with aspirin. The error bars represent the mean ± SEM (n = 3). (**C**,**D**) Confocal micrographs showing dose-dependent inhibition of cell proliferation marker Ki-67 in aspirin-treated cells, scale bars—50 µm. (**E**,**F**) Scatter plot representation of mean fluorescence intensity of Ki-67 marker. The mean fluorescence intensity was measured for 50 cells across five different random fields and quantified relative to the signal of DAPI. One-way ANOVA and Bonferroni post-test was used for statistical analysis, * *p* < 0.05, ** *p* < 0.01, *** *p* < 0.001. (**G**) Aspirin inhibits colony formation of GB-SCC cells. The error bars represent the mean ± SEM (n = 3). (**H**,**I**) The dose-dependent (0 mmol/L, 2.5 mmol/L, 5 mmol/L) effect of aspirin on distribution of cells in different cell cycle phases (n = 3). (**J**,**K**) Aspirin induces apoptosis and inhibits migration of (**L**) ITOC-03 and (**M**) ITOC-04 cells. The migration rate of GB-SCC cell lines was measured every 8 h in the presence and absence of aspirin. The error bars represent the mean ± SEM (n = 3). Student’s t test was used for statistical analysis, n = 3, * *p* < 0.05, ** *p* < 0.01, *** *p* < 0.001.

A significant dose- and time-dependent decrease in cell viability was observed in both ITOC-03 and ITOC-04 cells and the inhibitory effect was significantly higher in the AAM pathway down-regulated ITOC-04 cells (Figure 1A,B and Figure S1A,B). The highest concentration (10 mmol/L) was toxic with minimal cell viability; hence, further experiments were performed at 0, 2.5 and 5 mmol/L concentration. The IC_50_ value of aspirin-treated ITOC-03 and ITOC-04 cells at 24 h was 3.8419 and 3.5517, while that at 48 h was 2.5373 and 2.4593, respectively. Aspirin inhibited cell proliferation as measured using the Ki-67 proliferation marker (Figure 1C–F) and colony formation as visualized using crystal blue staining (Figure 1G). All these assays showed that aspirin had a significant dose- and time- dependent inhibitory effect on both cell lines, being more pronounced in the AAM pathway down-regulated ITOC-04 cell line. 

### 3.2. Cell Cycle Arrest, Apoptosis and Migration Affected with Aspirin Treatment

Aspirin resulted in a cell cycle perturbation with increase in the G0/G1 phase from 79% to 86% in ITOC-03 and from 61% to 82% in ITOC-04 in comparison to the untreated samples (Figure 1H,I). A dose-dependent increase in apoptosis as measured by the Annexin V stained population (Annexin V positive/PI negative) was seen in both ITOC-03 and ITOC-04 cells, with no significant difference between the two cell lines (Figure 1J,K). Apoptosis was also confirmed using Transmission Electron Microscopy where increased vacuolization was seen in both ITOC-03 and ITOC-04 cells while apoptotic bodies were observed only in the ITOC-04 cells. Discernible changes were not observed in mitochondria or nucleus of the cells treated with aspirin (Figure S2). The effect of aspirin on GB-SCC cell migration was studied with the scratch assay. Images were captured every 8 h over a period of 24 h. In both ITOC-03 and ITOC-04 cells, aspirin suppressed the cell migration, however, the effect was more pronounced in ITOC-04 cells (Figure 1L,M). The higher migratory potential of ITOC-04 cells was abrogated in a dose-dependent manner, especially at higher concentrations.

### 3.3. Inhibition of PLA2G6/COX/LOX and PLA2G6/NF-ĸB Pathway

**Figure 2:**
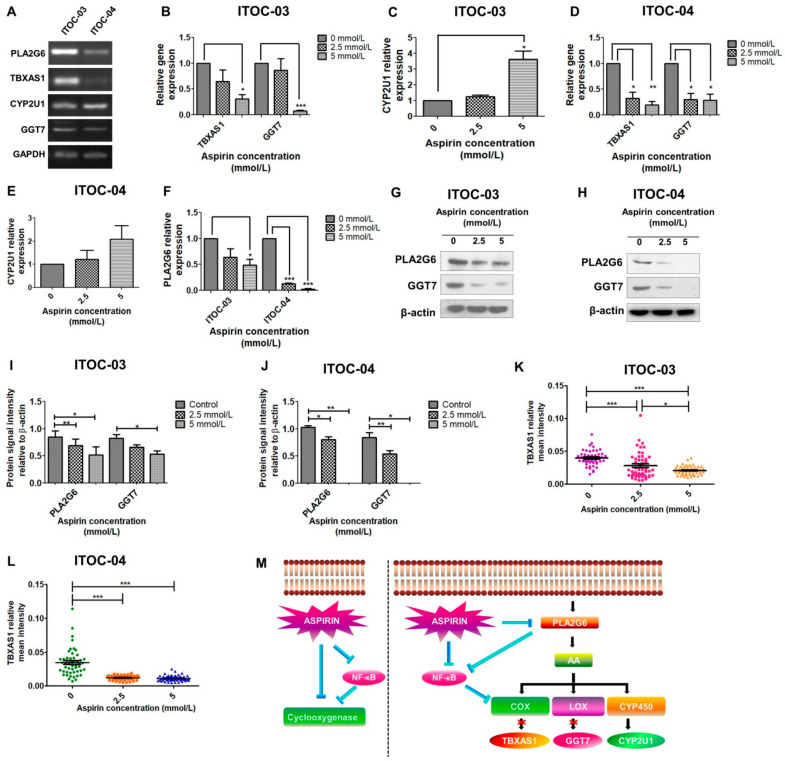
PLA2G6 inhibition by aspirin leads to down-regulation of AAM pathway components. (**A**) Expression of AAM pathway components PLA2G6, TBXAS1, GGT7 and CYP2U1 in GB-SCC cells as measured by RT-PCR. (**B**–**E**) Relative expression of TBXAS1, GGT7 and CYP2U1 in GB-SCC cells treated with aspirin as measured by qRT-PCR. The error bars represent the mean ± SEM (n = 3). Student’s t test was used for statistical analysis, * *p* < 0.05, ** *p* < 0.01, *** *p* < 0.001. (**F**) Relative expression of PLA2G6 in GB-SCC cells treated with aspirin as measured by qRT-PCR. The error bars represent the mean ± SEM (n = 3). Student’s t test was used for statistical analysis, * *p* < 0.05, *** *p* < 0.001. (**G**,**H**) Immunoblots depicting expression profile of PLA2G6 and GGT7 in aspirin-treated GB-SCC cells. β-actin used as endogenous control. (**I**,**J**) Quantification of PLA2G6 and GGT7 signal intensity relative to β-actin levels. The error bars represent the mean ± SEM (n = 3). Student’s t test was used for statistical analysis, * *p* < 0.05, ** *p* < 0.01. (**K**,**L**) Scatter plot representation of the mean fluorescence intensity of TBXAS1. The mean fluorescence intensity was measured for 50 cells across five different random fields and quantified relative to the signal of DAPI. One-way ANOVA and Bonferroni post-test were used for statistical analysis, * *p* < 0.05, *** *p* < 0.001. (**M**) Schematic representation of aspirin inhibition of arachidonic acid metabolism pathway. The left panel is demonstrating the known mechanism of action of aspirin on cyclooxygenase, and the right panel is showing the novel mechanism of action of aspirin in GB-SCC cells. NB: AA: arachidonic acid, COX: cyclooxygenase, LOX: lipoxygenase, CYP450: cytochrome P450.

We had previously reported the correlation between loss-of-function mutations in AAM pathway genes with better survival in GB-SCC patients [28]. Here, we evaluated the expression of the 10 AAM pathway genes mutated in GB-SCC - PLA2G3, PLA2G4E, PLA2G4F, PLA2G6, TBXAS1, PTGIS, GGT7, GPX7, CYP2U1, CYP2C19 in these two GB-SCC cell lines. Of these, 4 genes with significant expression in both cell lines (PLA2G6, TBXAS1, CYP2U1, GGT7) were chosen for further experiments (Figure 2A). We observed a dose-dependent inhibition of downstream components of the AAM pathway—TBXAS1 of the COX component and GGT7 of the LOX component (Figure 2B,D) and up-regulation of CYP2U1 of the CYP component of the AAM pathway (Figure 2C,E). The down-regulation of TBXAS1 was expected, as it is downstream of COX, a direct target of aspirin. However, the down-regulation of GGT7, a LOX component, suggested the involvement of an upstream component of the AAM pathway in aspirin-mediated inhibition. We further investigated the effect of aspirin on the upstream PLA2G6 gene belonging to the phospholipase family of the AAM pathway. Interestingly, we found that aspirin inhibits PLA2G6 expression in a concentration-dependent manner (Figure 2F). We further observed down-regulation of PLA2G6, GGT7 and TBXAS1 protein levels on treatment with aspirin (Figure 2G–L, Figures S3 and S4). 

With our observation of aspirin inhibiting PLA2G6, we further studied if in addition to the known direct effect of aspirin on NF-ĸB, aspirin could have an additive PLA2G6- mediated inhibitory effect on NF-ĸB (Figure 2M). We observed PLA2G6 siRNA knockdown (Figure S5) resulted in inhibition of NF-ĸB and two of the AAM pathway downstream components, COX (TBXAS1), which is also known to be mediated by NF-ĸB, as well as the LOX component (GGT7). Paradoxically, PLA2G6 knockdown resulted in increased expression of CYP2U1 which represents the CYP component of the AAM pathway.

### 3.4. Transcriptome Analysis Revealed Deregulation of AAM Pathway in ITOC-04 Cells

**Figure 3:**
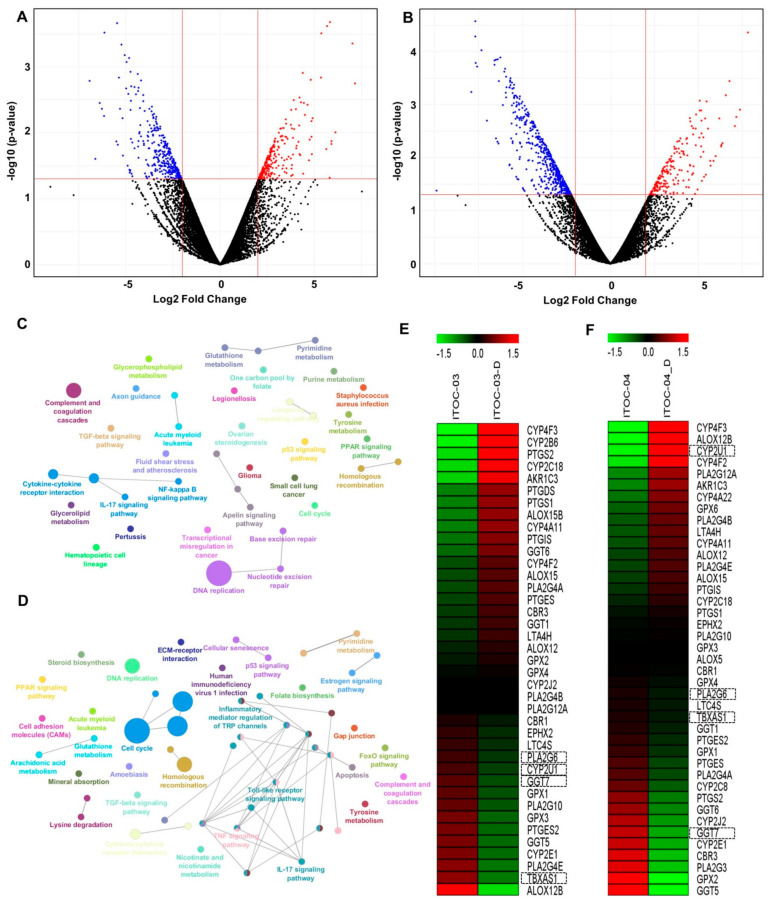
Differential gene expression and pathway enrichment of aspirin-treated GB-SCC cells. (**A**,**B**) Volcano plot highlighting significantly altered genes in aspirin-treated ITOC-03 and ITOC-04 cells, respectively. The scattered points on the plot represent genes. Blue dots denote down-regulated genes, red dots denote up-regulated genes, and black dots denote no significant change in gene expression on treatment with aspirin. Pathway enrichment analysis for aspirin-treated (**C**) ITOC-03 and (**D**) ITOC-04 cells using the ClueGO plugin in the cytoscape tool. Enriched pathways are displayed as nodes with different colors and only the most significant in the group are labelled. Multi-color nodes depict overlap between the genes. Heatmap depicting alterations in Arachidonic acid metabolism pathway genes in aspirin-treated (**E**) ITOC-03 and (**F**) ITOC-04 cells. Untreated cells are denoted as ITOC-03, ITOC-04 and aspirin-treated cells are denoted as ITOC-03_D and ITOC-04_D.

To further augment our understanding of the aspirin effect on GB-SCC cells, we performed global transcriptome profile of ITOC-03 and ITOC-04 cells after a 48 h treatment with aspirin at 5 mmol/L concentration (Figure 3A,B). The commonly deregulated pathways identified in ITOC-03 and ITOC-04 cells treated with aspirin included cell cycle, DNA replication, p53 signaling pathway, TGF-β signaling pathway, PPAR signaling pathway, cytokine-cytokine receptor interaction, IL-17 signaling pathway, and drug metabolism (Figure 3C,D). Interestingly, ITOC-04 showed deregulation of the AAM pathway. Additionally, ITOC-04 cells showed deregulation of ECM receptor interaction, cell adhesion molecules, FOXO signaling pathway, glutathione metabolism, cellular senescence, apoptosis, TNF signaling pathway, and Toll-like receptor signaling pathway. We further investigated the effect of aspirin on the AAM pathway (Figure 3E,F). On treatment with aspirin, down-regulation of the AAM pathway was seen in both cell lines as compared to the untreated cells. In corroboration with the in vitro findings, the down-regulation of PLA2G6, GGT7, and TBXAS1 was seen in both cell lines, however, CYP2U1 up-regulation was observed only in ITOC-04 aspirin-treated cells.

To validate our findings, we evaluated the expression levels of AAM pathway genes in the TCGA database of Head and Neck Squamous Cell Carcinoma (HNSCC). High expression of PLA2G6, GGT7, TBXAS1, and low expression of CYP2U1 were observed in the TCGA database of HNSCC tumors as compared to normal tissues (Figure S6A–D). Furthermore, corroborating our findings, the TCGA analysis showed significant positive correlation between PLA2G6 and GGT7 expression levels (Figure S6E). Co-expression of PLA2G6 was also observed with TBXAS1 and CYP2U1 (Figure S6F,G). 

### 3.5. Aspirin-Induced In Vivo Inhibition of PLA2G6 Leads to Synthetic Lethality in AAM Pathway Down-Regulated Xenografts

**Figure 4:**
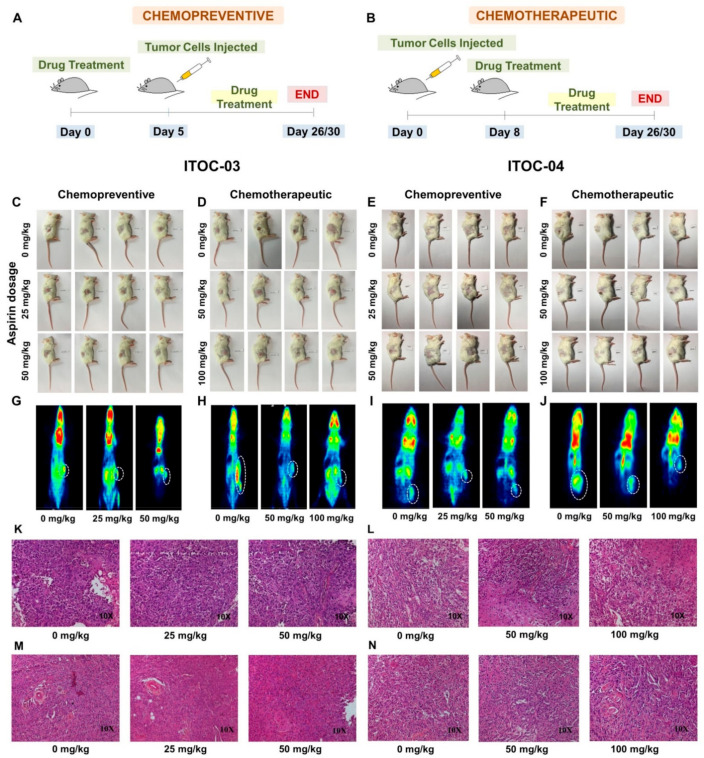
Aspirin suppresses the tumor growth in vivo. (**A**,**B**) Schematic representation of the in vivo chemopreventive and chemotherapeutic study in xenografted NOD-SCID mice. In chemopreventive studies, the aspirin treatment was initiated on Day 0 and continued until Day 5. The GB-SCC cells were injected on Day 5 and the aspirin treatment continued until 26 days for ITOC-03 cells and 30 days for ITOC-04 cells. In chemotherapeutic studies, the GB-SCC cells were injected on Day 0 and on initiation of the tumor formation on Day 8, the mice were subjected to aspirin treatment until 26 days for ITOC-03 cells and 30 days for ITOC-04 cells. (**C**–**F**) In vivo studies performed on xenografts of GB-SCC cells in presence and absence of aspirin. The mice were subjected to two different dosage regimens of aspirin; chemopreventive (0 mg/kg, 25 mg/kg, 50 mg/kg) and chemotherapeutic (0 mg/kg, 50 mg/kg, 100 mg/kg), n = 4/group. (**G**–**J**) Micro PET images of 18-FDG uptake in coronal planes for untreated and aspirin-treated xenografts of GB-SCC cells. (**K**–**N**) Microphotographs of H&E stained tumor xenografts captured at 10X magnification.

**Figure 5:**
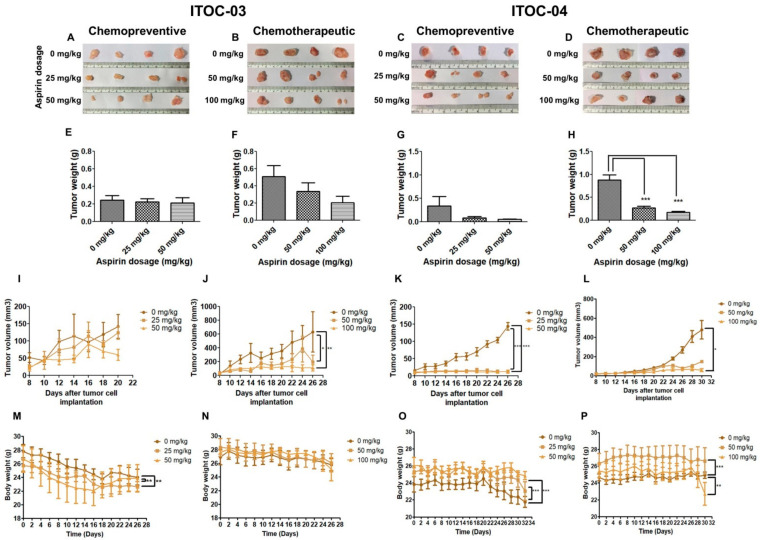
Chemopreventive and chemotherapeutic effect of aspirin on GB-SCC xenografts. (**A**–**D**) Photographs of ITOC-03 and ITOC-04 untreated and aspirin-treated tumor xenografts. The effect of different doses of aspirin on (**E**–**H**) Tumor weight (**I**–**L**) Tumor volume and (**M**–**P**) Body weight of mice. One-way ANOVA and Bonferroni post-test were used for statistical analysis, n = 4/group, * *p* < 0.05, ** *p* < 0.01, *** *p* < 0.001.

In vivo studies on xenografts of ITOC-03 and ITOC-04 cell lines in NOD-SCID mice (Figure 4A,B) showed prevention and growth suppression effect of aspirin, as assessed in two independent groups of mice (Figure 4C–F) by FDG PET-CT scan (Figure 4G–J), histological analysis which showed SCC morphology was recapitulated in the xenografts (Figure 4K–N) and with tumor size (Figure 5A–D) and weight (Figure 5E–H) measurements.

**Figure 6:**
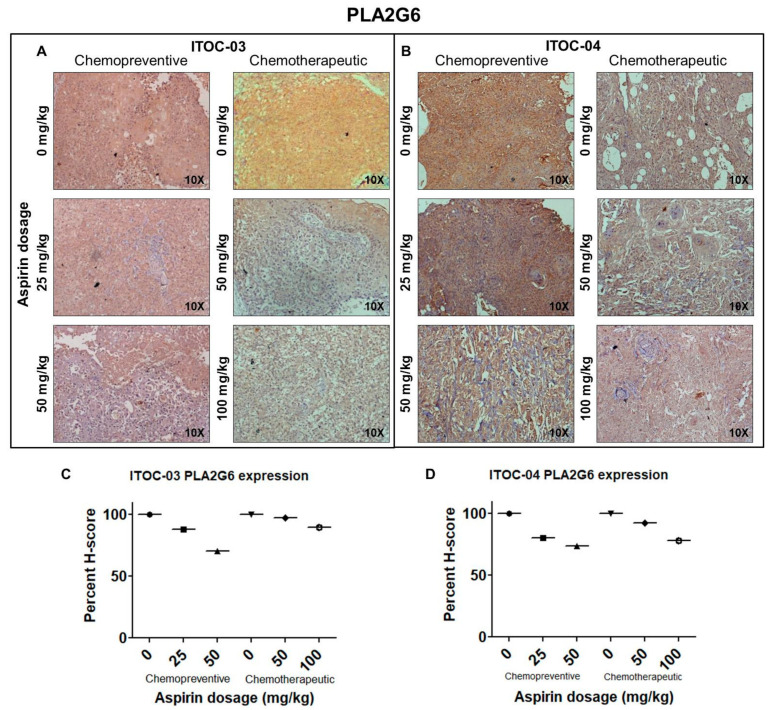
Immunohistochemical detection of PLA2G6 in xenografts generated from subcutaneous transplantation of GB-SCC cells, with or without aspirin. (**A**,**B**) PLA2G6 expression and (**C**,**D**) Percent H-score of PLA2G6 (n = 4), on treatment with different doses of aspirin; chemopreventive (0 mg/kg, 25 mg/kg, 50 mg/kg) and chemotherapeutic (0 mg/kg, 50 mg/kg, 100 mg/kg) in ITOC-03 and ITOC-04 derived xenografts, respectively.

It was observed that when aspirin was started 5 days prior to injection of 5 million GB-SCC cells and continued, it could prevent xenograft formation for the ITOC-04 SCC cells with the down-regulated AAM pathway at the lowest preventive dose of 25 mg/kg (Figure 5K) but for the ITOC-03 SCC cells, the preventive effect was only modest and with the highest preventive dose of 50 mg/kg (Figure 5I). In the chemotherapeutic group, aspirin administration started after formation of xenograft; it resulted in significant growth suppression in the ITOC-04 SCC xenograft with the down-regulated AAM pathway at the lowest therapeutic dose of 50 mg/kg (Figure 5L) but achieved similar levels of growth suppression for the ITOC-03 SCC xenograft only with the highest therapeutic dose level of 100 mg/kg (Figure 5J). There was no significant change in the body weight of the mice during the 26–30 day observation period in any of the treatment groups (Figure 5M–P). Immunohistochemical (IHC) analysis confirmed the down-regulation of PLA2G6 (Figure 6A–D), cell proliferation marker Ki-67 and AAM pathway components TBXAS1, GGT7, COX-2 and NF-ĸB (Figures S7–S11). The in vivo data corroborate with in vitro data wherein a more pronounced effect of aspirin was seen on ITOC-04-derived xenografts with the down-regulated AAM pathway as compared to ITOC-03. 

## 4. Discussion

Modulation of inflammation, a hallmark of cancer, could act as an effective therapy against cancer [12,13,30] with several studies supporting the role of aspirin or other anti-inflammatory drugs on cancer initiation, progression and metastasis [31,32,33]. However, the anti-inflammatory drugs mainly act on COX-1, COX-2 or LOX components of the AAM pathway. Targeting phospholipase (PLA2) enzymes, the key upstream substrates of the AAM pathway would inhibit the release of arachidonic acid and exert a potent anti-inflammatory action, thereby serving as a beneficial therapeutic intervention. Several studies have shown the role of secretory PLA2s and cPLA2s in tumorigenesis and cancer progression, however, few studies have demonstrated the role of iPLA2 in colon, pancreatic and ovarian cancer [34,35,36], and none in GB-SCC. Aspirin, a promising repositioning drug in oncology, is known to target the tumor promoting inflammation. In this study, we demonstrate a previously unrecognized mechanism of aspirin-mediated dual inhibition of PLA2G6 and COX-2 and a strong correlation between the inhibitory effect of aspirin with underlying down-regulation of the AAM pathway, suggesting synthetic lethality of aspirin in AAM pathway deficient cancer cells.

The in vitro and in vivo effect of aspirin observed in this study on GB-SCC cell proliferation, migration, apoptosis and on chemoprevention or growth suppression of xenografts is similar in nature and magnitude as previously reported in pre-clinical studies for other cancers [20,21,22,23,24,25,37,38]. Our findings show a pronounced effect of aspirin on ITOC-04 cells as compared to ITOC-03 cells, which is attributed to the key genetic differences in both the cell lines (Table S3). Based on X-ray crystallography studies of a complex of PLA2 and aspirin, it has been shown that aspirin is literally embedded in the hydrophobic environment of PLA2, occupying a favorable space in the specific binding site of PLA2 and its pharmacological effect may be through its interactions with PLA2 [39]. To the best of our knowledge, this is the first in vitro and in vivo study to show the effect of aspirin on the expression of PLA2G6 and its downstream COX and LOX components. 

Of the 3 components of the AAM pathway, genes of the COX and LOX components were down-regulated by aspirin but the CYP450 component gene CYP2U1 was up-regulated (Figure 2C,E). The inverse relation between expression of various phospholipases and CYP450 genes has been observed in earlier studies [40,41,42,43]. While this observation has not been fully explained, it has been postulated that AAM pathway inhibitors induce cell stress, which leads to AA release and some metabolite induces CYPIA1 activity [41]. Li et al. [43] have shown an inverse relation between PLA2G6 and CYP2C44 in hepatocellular carcinoma but the possible mechanism was not studied or discussed.

Further, in vivo studies showed aspirin mediated tumor growth suppression in GB-SCC xenograft models. In the present in vivo study, the term ‘chemopreventive’ is assigned to a group wherein aspirin was administered for a pre-defined period and thereafter tumor cells were injected with continued treatment of aspirin. Although, similar studies have been conducted using aspirin [44,45,46,47], the term ‘chemopreventive’ defined in xenograft tumor models is different from the true ‘chemopreventive’ effect described in human studies. Chemopreventive and chemotherapeutic doses ranged from 25 mg/kg–100 mg/kg. A low-dose regimen was followed considering the risk of gastrointestinal (GI) toxicity and renal failure associated on administration of aspirin, due to inhibition of prostaglandin which is involved in maintenance of renal function and also has cytoprotective effects in the GI tract [48,49]. An aspirin dosage of 25, 50 and 100 mg/kg in mice is equivalent to 2.03, 4.06 and 8.13 mg/kg in humans [22]. For a human weighing 60 kg, the chemopreventive aspirin dose extrapolated from our mice study would be as low as 120 mg to 250 mg daily, while the chemotherapeutic dose to prevent metastases would range between 250 mg to 500 mg daily. In the CAPP1 and CAPP2 trials for evaluating chemopreventive effect of aspirin in colorectal cancer, a 600 mg aspirin dose was assigned in Familial adenomatous polyposis (FAP) and Lynch syndrome individuals, respectively. CAPP1 trials showed a reduction in the largest polyp in individuals participating in the study over a year, while CAPP2 reported decreased cancer incidence in Lynch syndrome healthy mutation carriers. The current CAPP3 trial is comparing different doses of aspirin—100 mg, 300 mg, and 600 mg in Lynch syndrome cases [50,51,52,53]. The phase 3 Add-Aspirin trial is assessing the effect of aspirin on prevention of metastases and recurrence post radical therapy in gastro-oesophageal, colorectal, prostate and breast cancer using 100 mg and 300 mg aspirin per day [54,55]. Our preclinical data support the dose range of aspirin being investigated in the ongoing human trials in different cancers. 

The effect of aspirin was significantly greater in the ITOC-04 cell line with baseline down-regulation of the AAM pathway (Table S3) and may indicate synthetic lethality of aspirin in cancer cells with underlying partial deficiency in the AAM pathway. The phenomenon of synthetic lethality is being currently explored as an attractive approach for treatment of cancers [56]. We propose synthetic lethality-based chemoprevention for individuals whose genetic constitution causes partial insufficiency of some metabolic pathway which is tolerated due to alternate pathways. Complete inhibition of a partially functional pathway or the alternative metabolic pathway by widely used and safe drugs such as aspirin could induce death of the transformed dividing cells. Moreover, aspirin may exert chemopreventive effect at lower and less toxic doses in some genetic backgrounds. 

## 5. Conclusions

In summary, this study provides first experimental evidence of aspirin inhibiting iPLA2 and two major components of the AAM pathway, with possible synthetic lethality in cancer cells with down-regulated AAM pathway. These mechanistic insights and chemopreventive effect of aspirin in the dose range equivalent to the ongoing human trials could help in designing chemoprevention trials of AAM pathway inhibitors, possibly precision prevention studies adapted to baseline AAM pathway status in high risk individuals, and using appropriate biomarkers related to AAM pathway or its downstream genes.

## Data Availability

The data are available from the corresponding author on reasonable request.

## Supplementary Materials

The following supporting information can be downloaded at: https://www.mdpi.com/article/10.3390/cells11010123/s1, Supplementary Materials & Methods, Figure S1: Aspirin treatment leads to inhibition of cell viability, Figure S2: Detection of apoptosis induced by aspirin treatment of GB-SCC cells using TEM, Figure S3: Whole Western blot membranes for Figure 3G, Figure S4: Whole Western blot membranes for Figure 3H, Figure S5: Down-regulation of PLA2G6 expression following siRNA knockdown in GB-SCC cells, Figure S6: Expression of AAM pathway genes in normal and HNSCC tissues, Figure S7: Immunohistochemical detection of Ki-67 in xenografts generated from subcutaneous transplantation of GB-SCC cells, with or without aspirin, Figure S8: Immunohistochemical detection of TBXAS1 in xenografts generated from subcutaneous transplantation of GB-SCC cells, with or without aspirin, Figure S9: Immunohistochemical detection of GGT7 in xenografts generated from subcutaneous transplantation of GB-SCC cells, with or without aspirin, Figure S10: Immunohistochemical detection of COX-2 in xenografts generated from subcutaneous transplantation of GB-SCC cells, with or without aspirin, Figure S11: Immunohistochemical detection of NF-ĸB in xenografts generated from subcutaneous transplantation of GB-SCC cells, with or without aspirin, Table S1: siRNA sequences, Table S2: Primer sequences for qPCR, Table S3: Key genetic and aspirin treatment induced differences in ITOC-03 and ITOC-04 cell lines.
