# Supplementary material for: Aspirin Inhibition of Group VI Phospholipase A2 Induces Synthetic Lethality in AAM Pathway Down-Regulated Gingivobuccal Squamous Carcinoma
Source: Cells. 2021 Dec 30;11(1):123. doi: 10.3390/cells11010123 (PMC8750243; doi:10.3390/cells11010123)
Supplement: Supplementary file 1 [file cells-11-00123-s001.zip › cells-1492331-supplementary.pdf]

Supplementary Materials: Aspirin inhibition of group VI phospholipase A2 induces synthetic lethality in AAM pathway down-regulated gingivobuccal squamous carcinoma

Kshama Pansare[1], Bhabani Mohanty[2], Ranjeeta Dhotre[1], Aafrin M Pettiwala[1], Saili Parab[3], Neha Gupta[1], Poonam Gera[1,3], Nilesh Gardi[4,-5], Rucha Dugge[1], Priyanka Sahu[1], Ruby Alhans[1], Pradnya Kowtal[1,5,6], Pradip Chaudhari[2,5] and Rajiv Sarin[1,5,6*]

Supplementary Materials & Methods

Immunoblotting

Membranes were blocked with 5% milk or BSA in TBST and incubated with primary antibody for 1 hour at room temperature or overnight at 4°C; PLA2G6 (Rabbit polyclonal, Thermo Scientific, Rockford, USA, PA5-26936, 1:1000), GGT7 (Rabbit polyclonal, Abcam, MA, USA, ab80903, 1:1000,), β-actin (Mouse monoclonal, Abcam, MA, USA, ab6276, 1:1000). HRP linked secondary antibody (Anti-rabbit, GE Healthcare, Darmstadt, Germany, NA934, and Anti-mouse, Cell Signaling, MA, USA, 7076) was incubated for 1 hour at room temperature followed by chemiluminescent detection using SuperSignal West Pico Chemiluminescent Substrate (ThermoScientific, Carlsbad, USA).

RNA sequencing

RNA-seq analysis was performed as described earlier [29]. Functional enrichment analysis of significantly altered genes was carried out using ClueGO plug-in from cytoscape tool. Kyoto Encyclopedia of Genes and Genomes (KEGG) pathway was used for enrichment analysis. Heatmap for Arachidonic acid metabolism pathway related genes were generated using log transformed TPM (transcript per million) values and visualized using Multi Experiment Viewer software version 4.9. Comparison with TCGA database analysis was performed using UACLAN data portal (http://ualcan.path.uab.edu/) and cBioPortal (https://www.cbioportal.org/).

Mouse xenograft assay and PET imaging

In case of chemopreventive studies, mice were randomly subjected to 0 mg/kg, 25 mg/kg and 50 mg/kg aspirin dose 5 days prior to injection of tumor cell lines. 5 x 10^6 cells were injected subcutaneously in male in-house bred NOD-SCID mice and the aspirin treatment was continued until 20 and 26 days in ITOC-03 and -04 derived tumors, respectively. For chemotherapeutic studies, 5 x 10^6 cells were injected subcutaneously in male in-house bred NOD-SCID mice. On formation of tumor the mice were randomly allocated to three different groups and subjected to aspirin treatment of 0 mg/kg, 50 mg/kg and 100 mg/kg until 26 and 30 days in ITOC-03 and -04 derived tumors, respectively. The mice were housed in sterile pathogen-free environments and observed daily for the appearance of tumors. Tumor volume and body weight was measured on a regular basis. PET studies were done on aspirin treated and untreated NOD-SCID mice as described earlier [29]. On completion of treatment, mice were sacrificed and tumors were fixed in 10% formalin, embedded in paraffin, sectioned with microtome (5 µm) and stained with haematoxylin and eosin (H&E). Slides were examined for morphological details under light microscope and images captured using Axio Imager.Z1 upright microscope (Carl Zeiss, Gottingen, Germany) at 10X magnification.

Immunohistochemical analysis

Formalin fixed paraffin embedded tissue sections were deparaffinized with xylene and graded alcohol. Heat induced epitope retrieval using Tris or Citrate buffer was followed by 30 minutes incubation with blocking buffer. Slides were incubated with primary antibodies for 1 hour at room temperature or overnight at 4°C; horseradish peroxidase polymer based detection kit (Envision Plus, Dako, Glostrup, Denmark) for 30 minutes and DAB substrate for 1-3 minutes. Haematoxylin was used as counterstain and slides mounted using DPX mountant. Tumor tissue sections of mice were stained for Ki-67 (Cell Signaling, MA, USA, 9449, 1:300), NF-κB (Cell Signaling, MA, USA, 6956, 1:600), GGT7 (Abcam, MA, USA, ab80903, 1:100), PLA2G6 (Thermo Scientific, Rockford, USA, PA5-26936, 1:50), COX-2 (Cell Signaling, MA, USA, 12282, 1:100) and TBXAS1 (Abcam, MA, USA, ab119057, 1:200). Intensity of staining was assessed by pathologist

and percentage scoring was based on nuclear or cytoplasmic staining of respective markers. The intensity of staining was defined as 1+: weak, 2+: moderate and 3+: strong. The H-score was calculated based on intensity and percentage of the scoring.

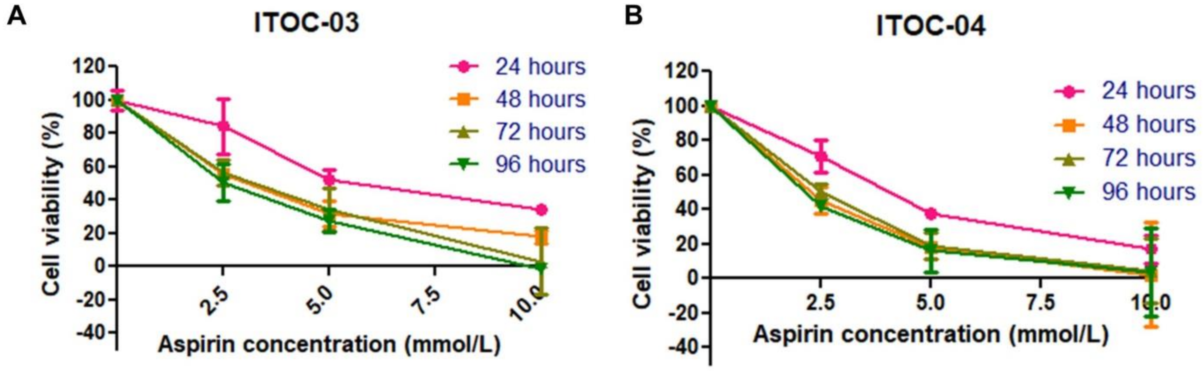

Figure S1. Aspirin treatment leads to inhibition of cell viability. (A-B) Cell viability assay showing dose- (0, 2.5, 5 and 10 mmol/L) and time-dependent (24 hours, 48 hours, 72 hours and 96 hours) inhibition of ITOC-03 and ITOC-04 cells on treatment with aspirin. The error bars represent the mean ± SEM (n=3).

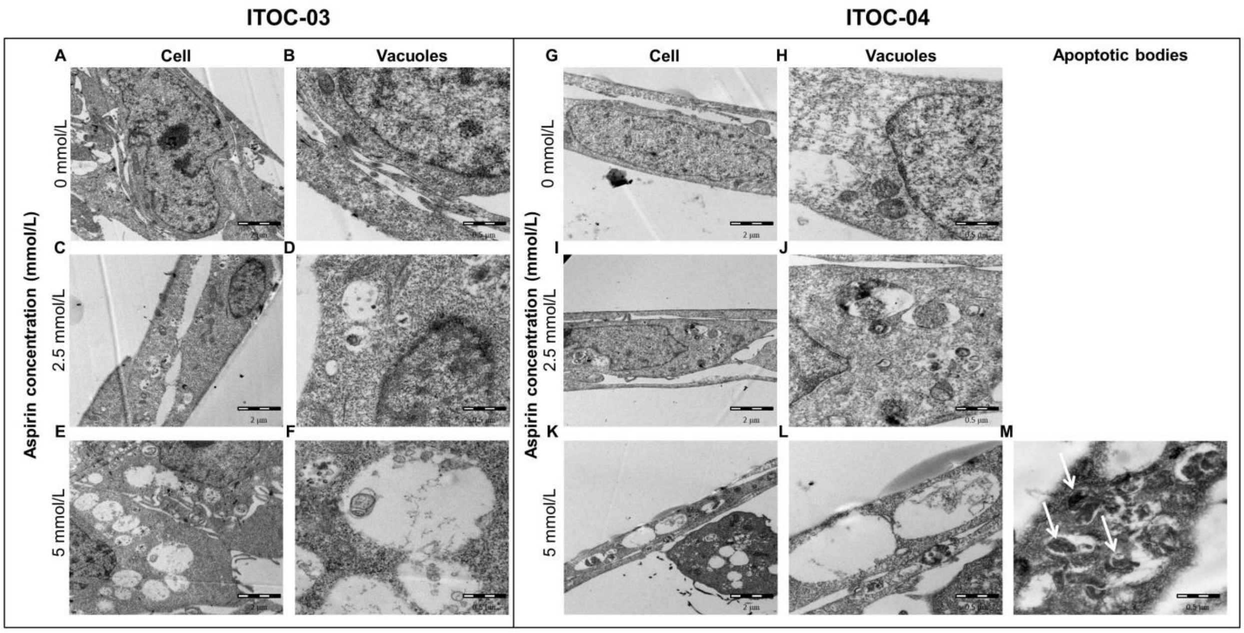

Figure S2. Detection of apoptosis induced by aspirin treatment of GB-SCC cells using TEM (A-F) ITOC-03 and (G-M) ITOC-04 cells were treated with 2.5 mmol/L and 5 mmol/L aspirin and analyzed by TEM. NB: White arrows indicate apoptotic bodies seen in ITOC-04 cells on treatment with 5 mmol/L aspirin.

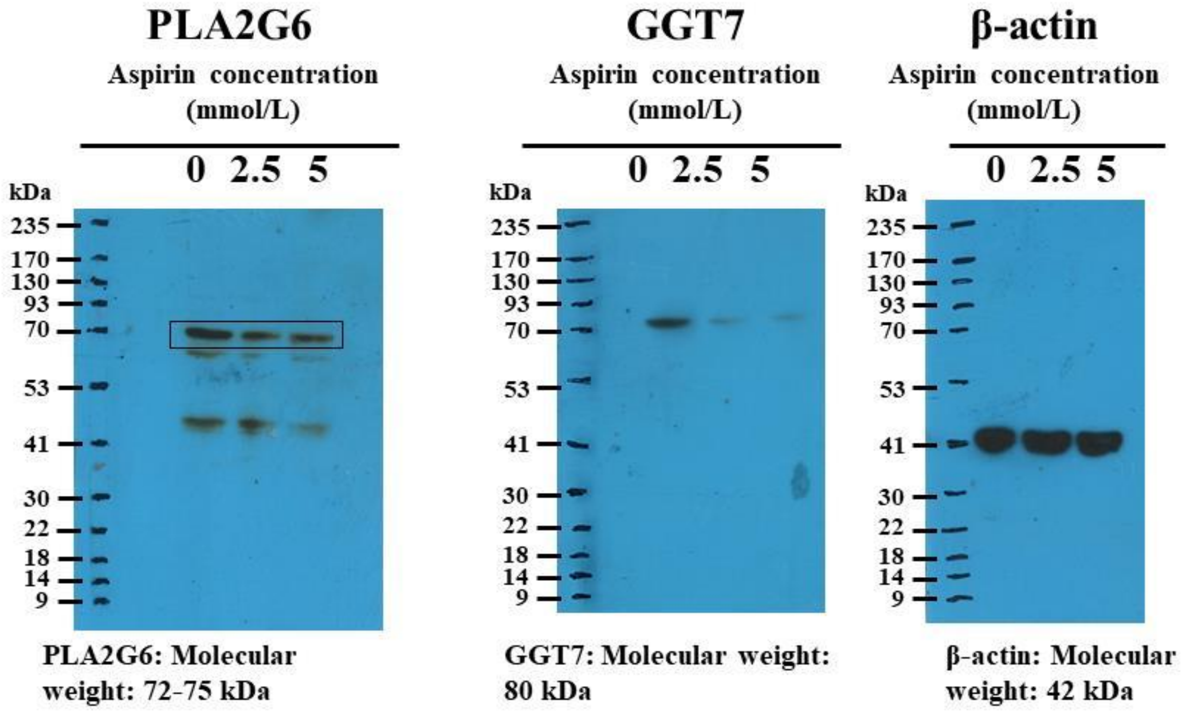

Figure S3. Whole Western blot membranes for Figure 3G.

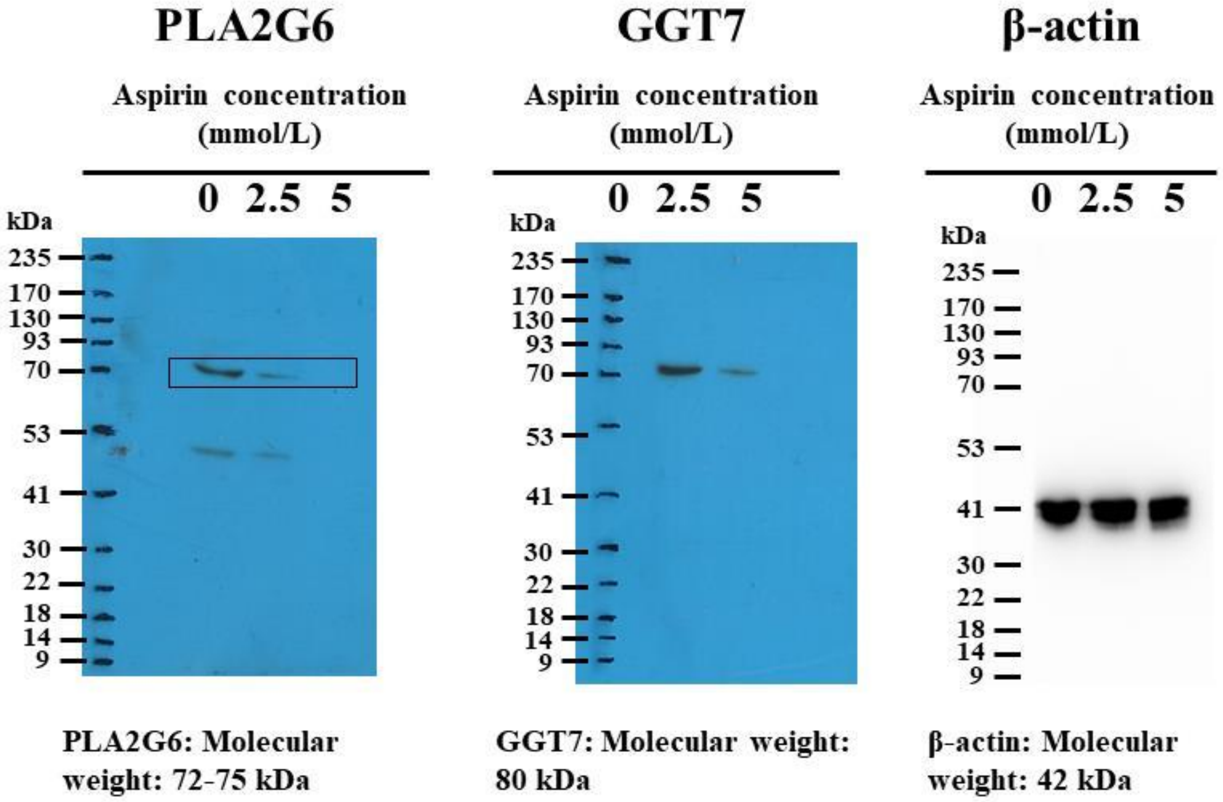

Figure S4. Whole Western blot membranes for Figure 3H.

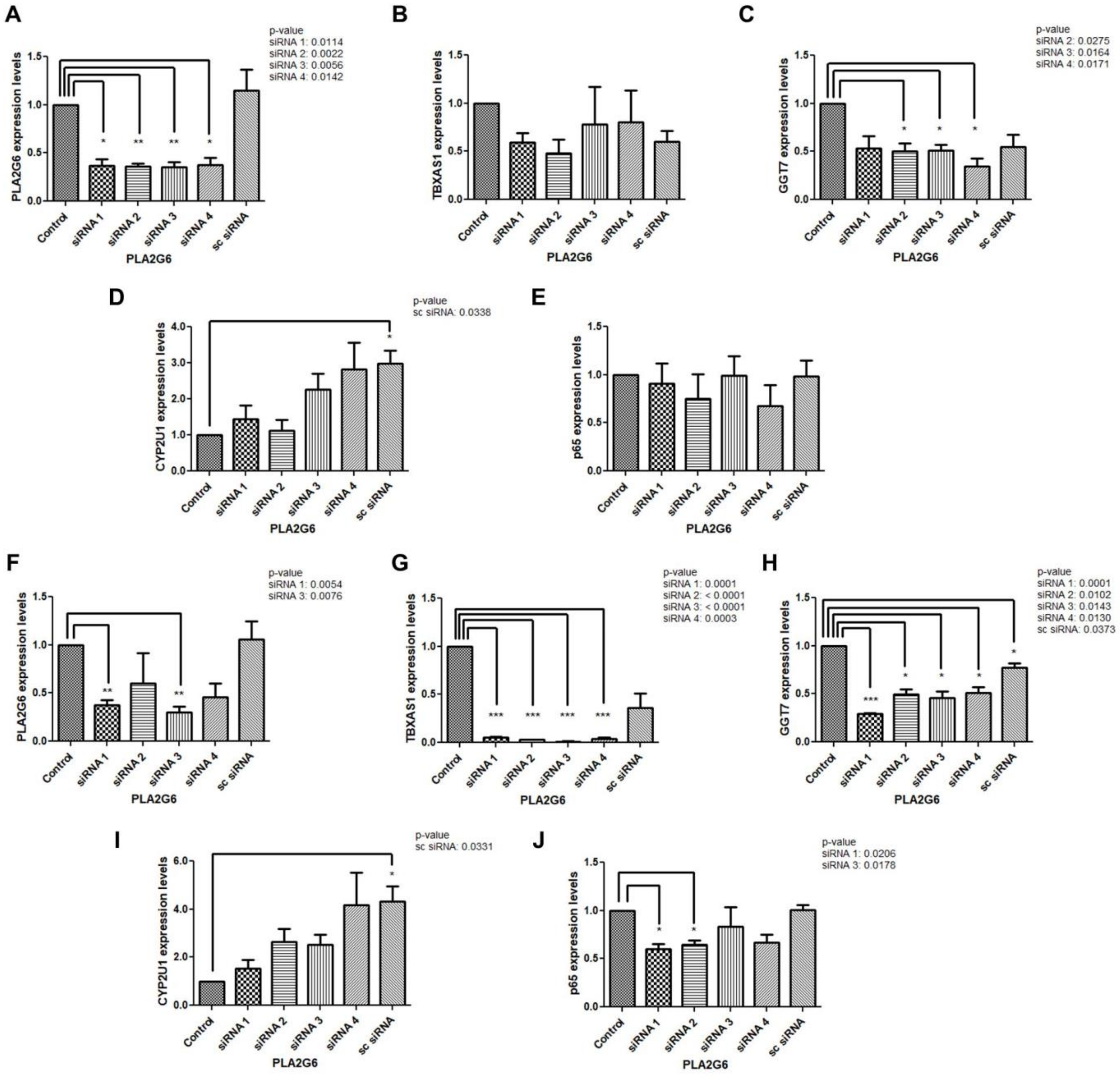

Figure S5. Down-regulation of PLA2G6 expression following siRNA knockdown in GB-SCC cells. The effect of siRNA knockdown on expression levels of PLA2G6, TBXAS1, GGT7, CYP2U1 and p65 in (A-E) ITOC-03 and (F-J) ITOC-04 cells as measured by qRT-PCR. The error bars represent the mean ± SEM (n=3). Student's t test was used for statistical analysis, *P < 0.05, **P < 0.01, ***P < 0.001.

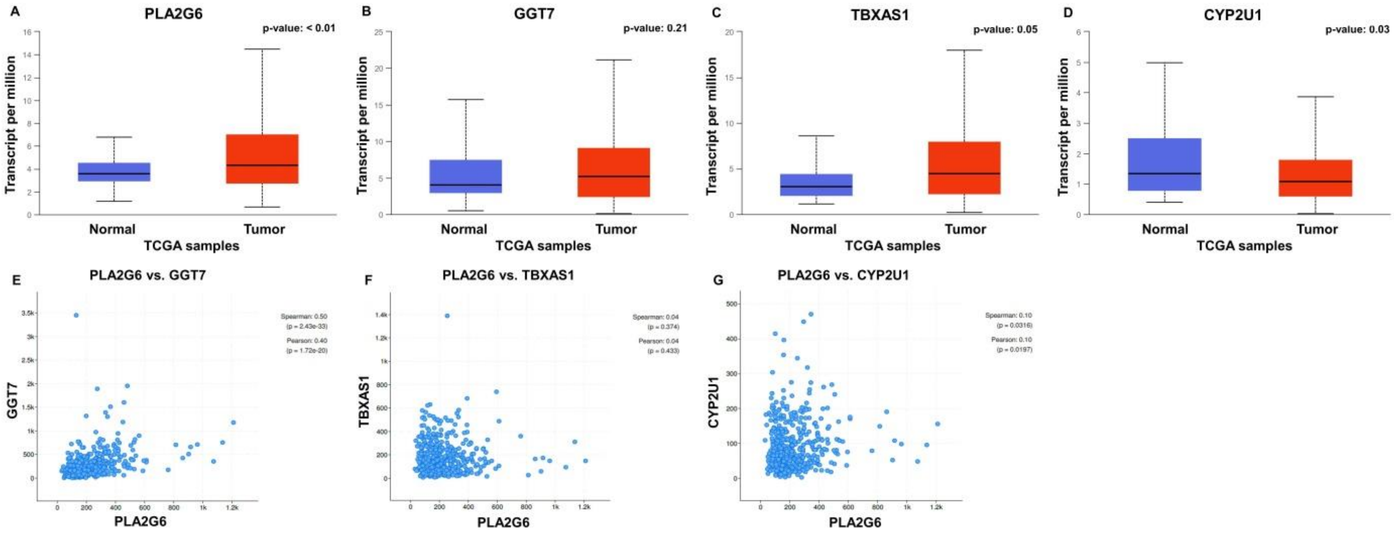

Figure S6. Expression of AAM pathway genes in normal and HNSCC tissues. (A-D) Validation of RNA-seq findings of PLA2G6, GGT7, TBXAS1 and CYP2U1 was performed using gene expression profiling data of 520 HNSCC tissues and 44 normal tissues in TCGA database (http://ualcan.path.uab.edu/). (E-G) Correlation of PLA2G6, GGT7, TBXAS1 and CYP2U1 genes in TCGA database of HNSCC tissues (https://www.cbioportal.org/).

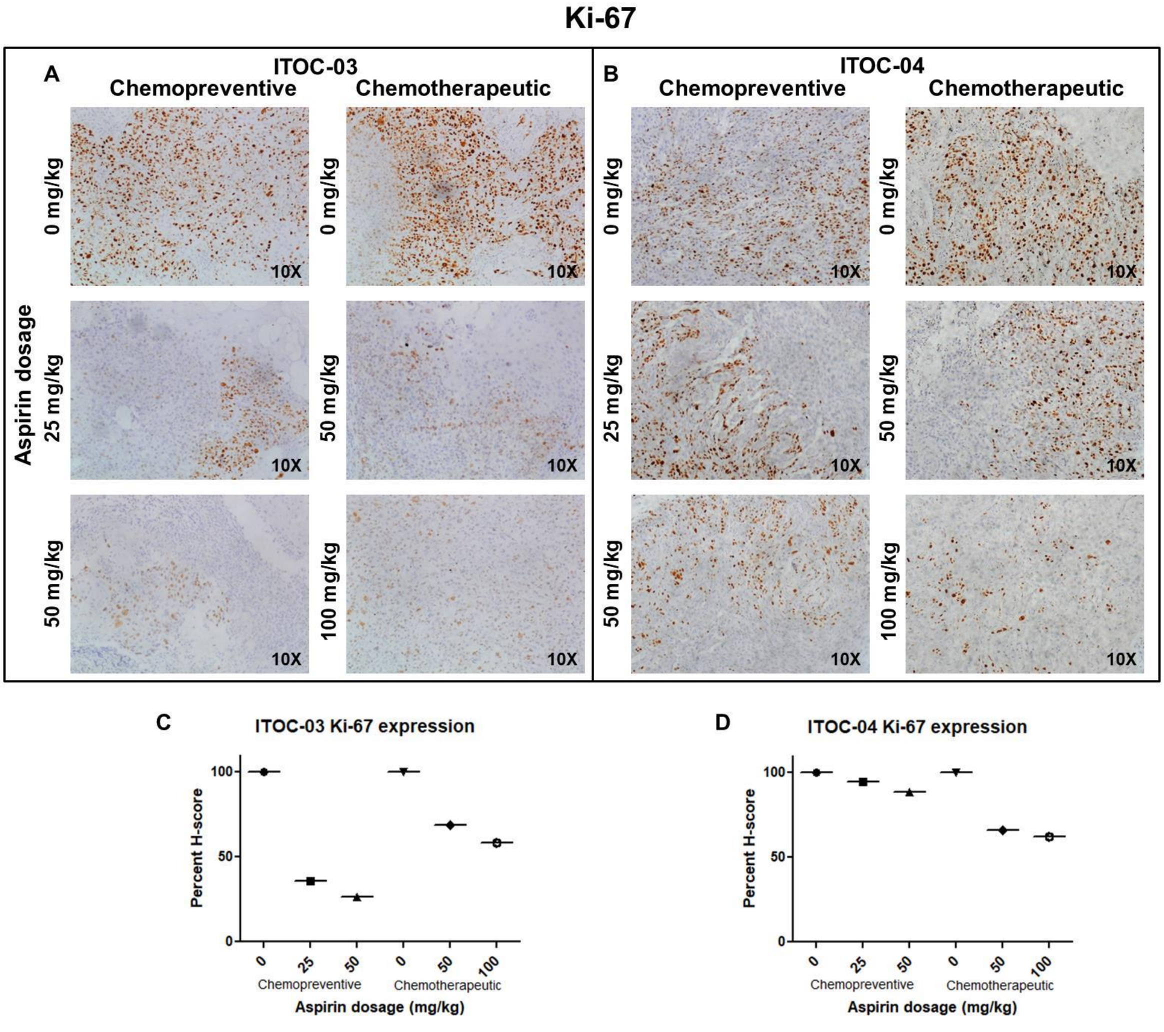

Figure S7. Immunohistochemical detection of Ki-67 in xenografts generated from subcutaneous transplantation of GB-SCC cells, with or without aspirin. (A-B) Ki-67 expression and (C-D) Percent H-score of Ki-67 (n=4), on treatment with different doses of aspirin; chemopreventive (0 mg/kg, 25 mg/kg, 50 mg/kg) and chemotherapeutic (0 mg/kg, 50 mg/kg, 100 mg/kg) in ITOC-03 and ITOC-04 derived xenografts respectively.

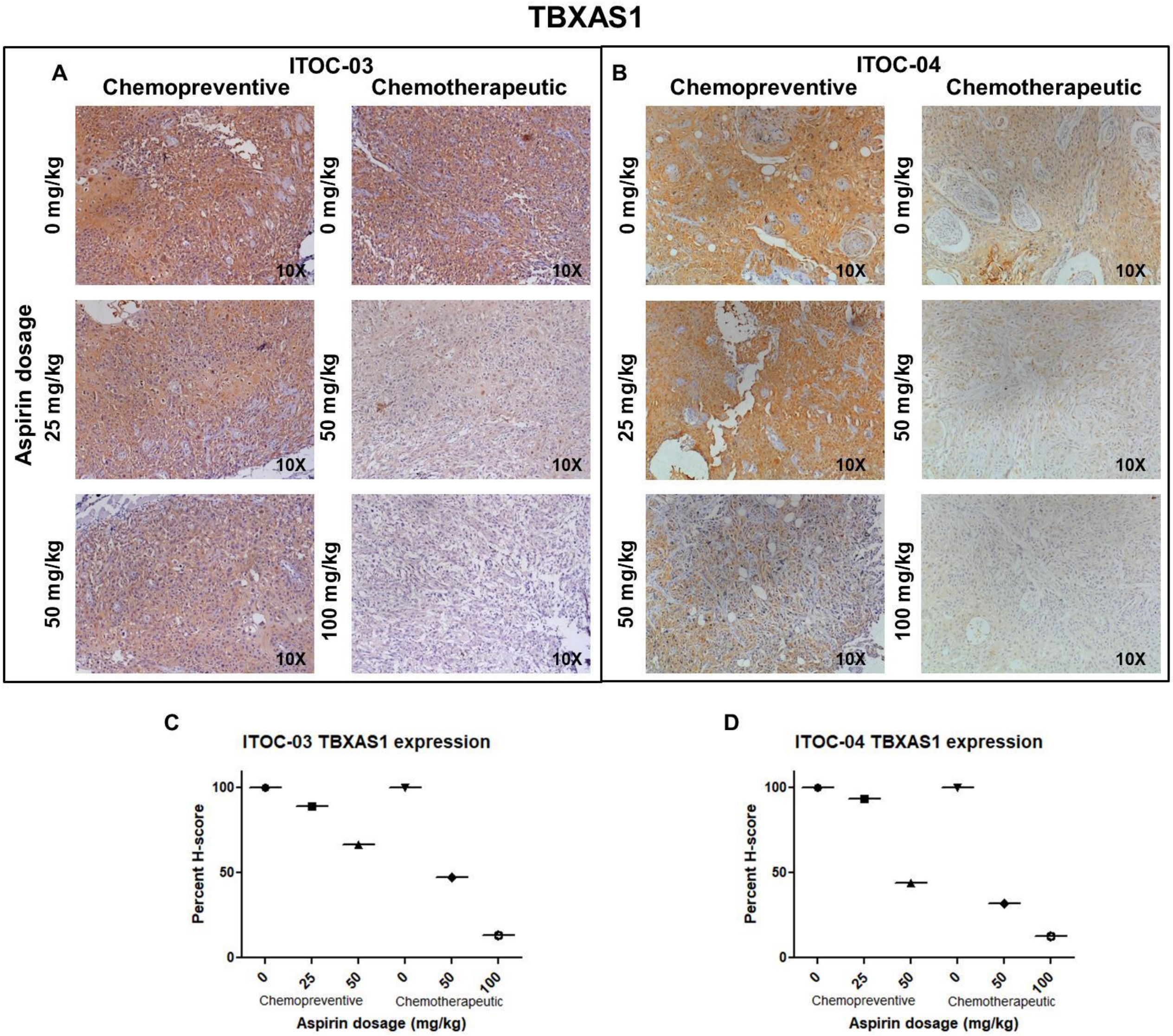

Figure S8. Immunohistochemical detection of TBXAS1 in xenografts generated from subcutaneous transplantation of GB-SCC cells, with or without aspirin. (A-B) TBXAS1 expression and (C-D) Percent H-score of TBXAS1 (n=4), on treatment with different doses of aspirin; chemopreventive (0 mg/kg, 25 mg/kg, 50 mg/kg) and chemotherapeutic (0 mg/kg, 50 mg/kg, 100 mg/kg) in ITOC-03 and ITOC-04 derived xenografts respectively.

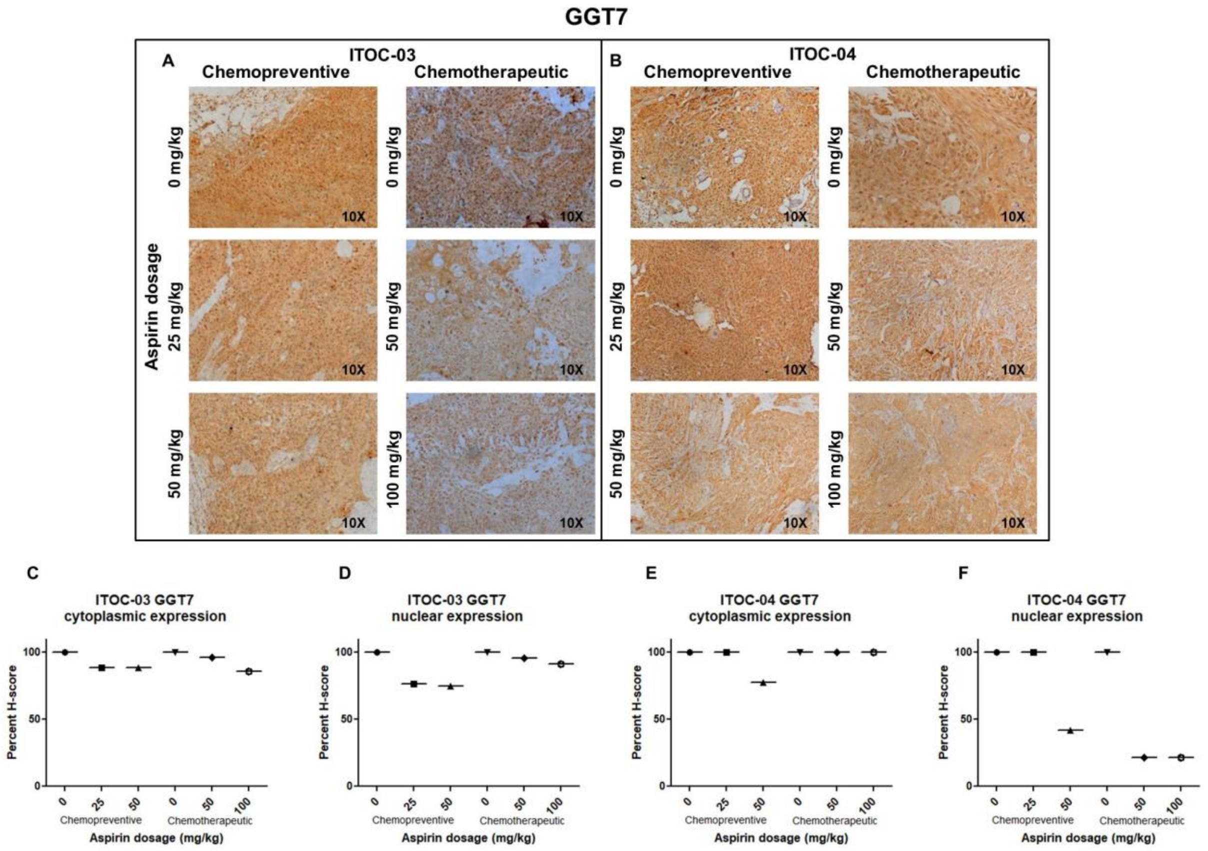

Figure S9. Immunohistochemical detection of GGT7 in xenografts generated from subcutaneous transplantation of GB-SCC cells, with or without aspirin. (A-B) GGT7 expression and (C-F) Percent H-score of GGT7 (n=4), on treatment with different doses of aspirin; chemopreventive (0 mg/kg, 25 mg/kg, 50 mg/kg) and chemotherapeutic (0 mg/kg, 50 mg/kg, 100 mg/kg) in ITOC-03 and ITOC-04 derived xenografts respectively.

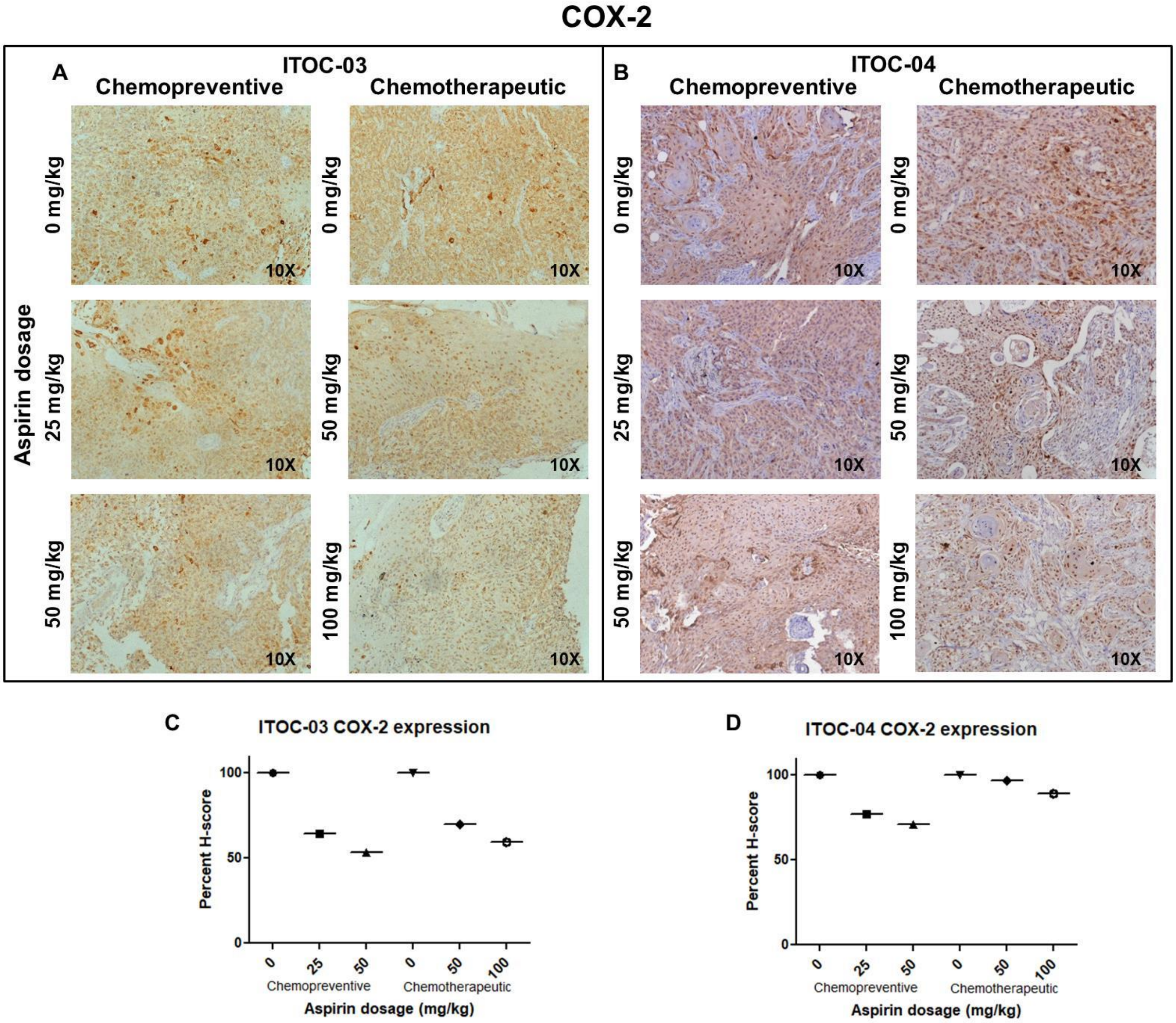

Figure S10. Immunohistochemical detection of COX-2 in xenografts generated from subcutaneous transplantation of GB-SCC cells, with or without aspirin. (A-B) COX-2 expression and (C-D) Percent H-score of COX-2 (n=4), on treatment with different doses of aspirin; chemopreventive (0 mg/kg, 25 mg/kg, 50 mg/kg) and chemotherapeutic (0 mg/kg, 50 mg/kg, 100 mg/kg) in ITOC-03 and ITOC-04 derived xenografts respectively.

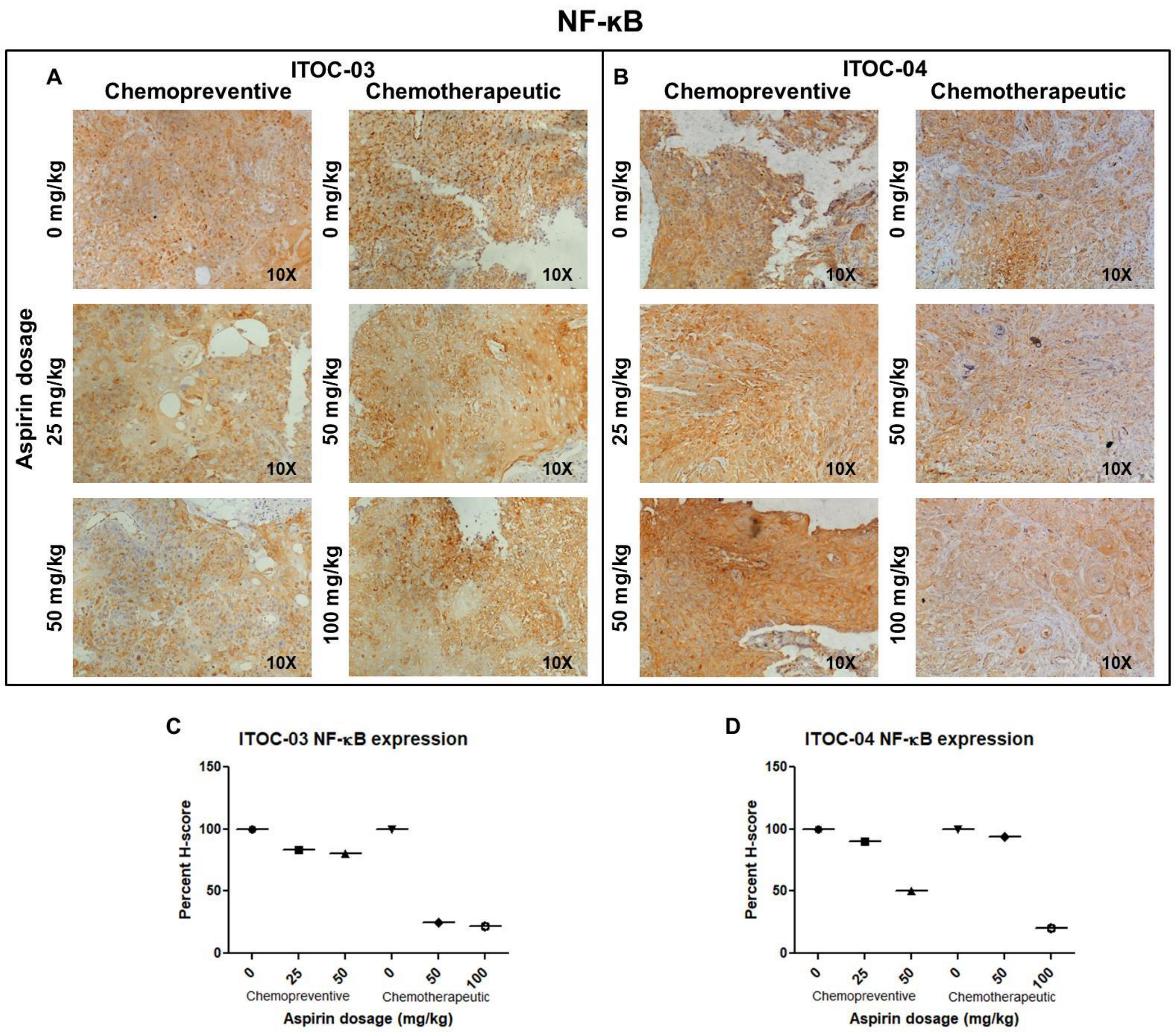

Figure S11. Immunohistochemical detection of NF-κB in xenografts generated from subcutaneous transplantation of GB-SCC cells, with or without aspirin. (A-B) NF-κB expression and (C-D) Percent H-score of NF-κB (n=4), on treatment with different doses of aspirin; chemopreventive (0 mg/kg, 25 mg/kg, 50 mg/kg) and chemotherapeutic (0 mg/kg, 50 mg/kg, 100 mg/kg) in ITOC-03 and ITOC-04 derived xenografts respectively.

Table S1. siRNA sequences.

siRNA	Sequence
PLA2G6 siRNA 1	UCAAGGCCCUCAUCGUGUU
PLA2G6 siRNA 2	GUAAGUCCAUGGCCUACAU
PLA2G6 siRNA 3	CGAGAUCCAUGAGUACAAU
PLA2G6 siRNA 4	AGAUGGAUGUCACCGACUA
scrambled siRNA Pool / Non-targeting pool	UGGUUUACAUGUCGACUAA
	UGGUUUACAUGUUGUGUGA
	UGGUUUACAUGUUUUCUGA
	UGGUUUACAUGUUUUCCUA

Table S2. Primer sequences for qPCR.

Genes	Forward Primer	Reverse Primer
**PLA2G6**	GCTCCTACTTACTTCCGACC	ACACAGGTCACAGGCACTTG
**TBXAS1**	AGCAGGTGTTGGTTGAGAAC	AGGACTGAAAGCAGACATCAGG
**CYP2U1**	TACATAGAGACCCAGCCA	GCATTAGGCTCACAAACAT
**GGT7**	TGGAACCTACCTCGCTCT	GGACCCAGGATAAGACAT
**p65**	AGCCCTATCCCTTTACGTCAT	TTCATCATCAAACTGCAGCTGCA
**β-ACTIN**	AAGAGAGGCATCCTCACCCT	TACATGGCTGGGGTGTTGAA

Table S3. Key genetic and aspirin treatment induced differences in ITOC-03 and ITOC-04 cell lines.

Key features	ITOC-03	ITOC-04
Primary and xenografted tumor	Derived from moderately differentiated SCC of GBS in a male chronic tobacco chewer.<br><br>Xenograft: Histopathologically confirmed SCC	Derived from poorly differentiated SCC of GBS in a male chronic tobacco chewer.<br><br>Xenograft: Histopathologically confirmed SCC
Key genes with loss of function mutation	CASP8, NOTCH1, NSD1, TP53,UNC13C - all with *pathogenic missense mutations	FAT3 (Stopgain), FAT1 (stopgain, *pathogenic missense), NOTCH1 (Splice site), TP53 (Stopgain), PCLO (*pathogenic missense)
Arachidonic Acid Metabolism (AAM) pathway	Not deregulated	Down-regulated AAM pathway
PLA2G6	↓↓ by Aspirin	↓↓↓↓ by Aspirin
NF-κB	↓↓ by Aspirin<br>↓↓ PLA2G6 siRNA	↓↓↓↓ by Aspirin<br>↓↓↓↓ by PLA2G6 siRNA
COX component - TBXAS1	↓↓ by Aspirin<br>↓↓ by PLA2G6 siRNA	↓↓↓↓ by Aspirin<br>↓↓↓↓ by PLA2G6 siRNA
LOX component - GGT7	↓↓ by Aspirin<br>↓↓ by PLA2G6 siRNA	↓↓↓↓ by Aspirin<br>↓↓↓↓ by PLA2G6 siRNA
CYP450 component - CYP2U1	↑↑ by Aspirin<br>↑↑ by PLA2G6 siRNA	↑↑ by Aspirin<br>↑↑ by PLA2G6 siRNA
Chemopreventive & tumor suppression effect of Aspirin	Modest	Marked
